# In vivo effects of the alpha-synuclein misfolding inhibitor minzasolmin supports clinical development in Parkinson’s disease

**DOI:** 10.1038/s41531-023-00552-7

**Published:** 2023-07-17

**Authors:** Diana L. Price, Asma Khan, Rachel Angers, Alvaro Cardenas, Maria Key Prato, Massimo Bani, Douglas W. Bonhaus, Martin Citron, Anja-Leona Biere

**Affiliations:** 1 Neuropore Therapies, Inc., San Diego, CA USA; 2 UCB Biopharma SPRL, Braine l’Alleud, Belgium

**Keywords:** Parkinson's disease, Pharmacology

## Abstract

Direct targeting of alpha-synuclein (ASYN) has emerged as a disease-modifying strategy for Parkinson’s disease and other synucleinopathies which is being approached using both small molecule compounds and ASYN-targeted biologics. Minzasolmin (UCB0599) is an orally bioavailable and brain-penetrant small molecule ASYN misfolding inhibitor in clinical development as a disease-modifying therapeutic for Parkinson’s disease. Herein the results of preclinical evaluations of minzasolmin that formed the basis for subsequent clinical development are described. Pharmacokinetic evaluations of intraperitoneal 1 and 5 mg/kg minzasolmin in wildtype mice revealed parallel and dose-proportional exposures in brain and plasma. Three-month administration studies in the Line 61 transgenic mouse model of PD were conducted to measure ASYN pathology and other PD-relevant endpoints including markers of CNS inflammation, striatal DAT labeling and gait. Reductions in ASYN pathology were correlated with improved aspects of gait and balance, reductions in CNS inflammation marker abundance, and normalized striatal DAT levels. These findings provide support for human dose determinations and have informed the translational strategy for clinical trial design and biomarker selection for the ongoing clinical studies of minzasolmin in patients living with early-stage Parkinson’s disease (ClinicalTrials.gov ID: NCT04658186; EudraCT Number 2020–003265).

## Introduction

The misfolding and aggregation of alpha-synuclein (ASYN) are key pathological drivers of Parkinson’s disease (PD) and related synucleinopathies^1,2^. ASYN is a 14 kDa presynaptic protein that plays a role in synaptic vesicle recycling. Evidence suggests that under normal physiological conditions ASYN is an intrinsically disordered protein existing in a predominantly monomeric form in dynamic equilibrium with non-propagating and propagating multimeric forms^3–7^. During the development of synucleinopathy diseases, this balance is proposed to shift towards to a preponderance of misfolded multimeric forms assembling in a pathological manner. Intraneuronal inclusions of aggregated ASYN, termed Lewy bodies, are the pathological hallmark of PD and are present in patients affected by sporadic and genetically inherited forms of the disease^1^.

**Fig. 1:**
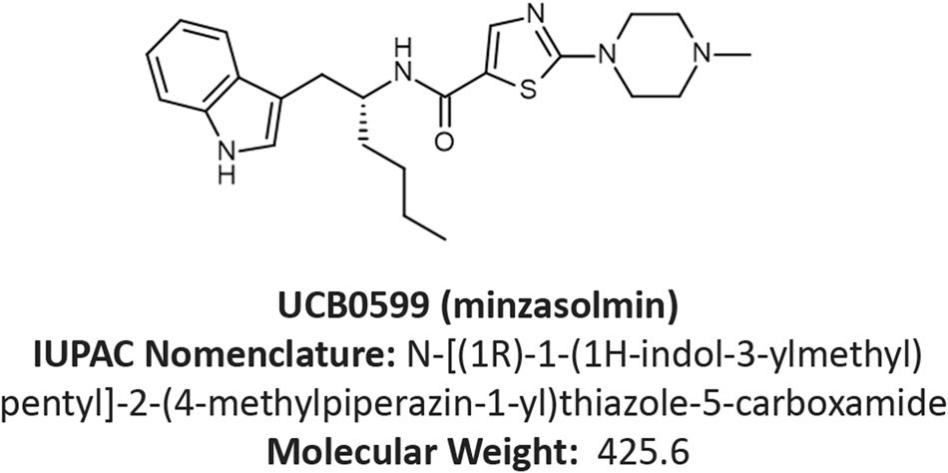
Structure of minzasolmin (UCB0599): a clinical stage small molecule advancing through development as a putative therapeutic for Parkinson’s disease. The structure, IUPAC nomenclature and molecular weight of minzasolmin (International Patent Publication Number WO 2015/116663 A1). The rendering was generated by Biovia Draw 2020 version 20.1.

Single point mutations in the SNCA gene cause PD in an autosomal dominant manner^8,9^. Alpha-synuclein constructs expressing these mutations can influence steps associated with aggregation in vitro^10–15^. Duplications and triplications of the SNCA locus are also sufficient to cause PD^16^ and a gene dosage effect has been described with patients expressing the highest levels of ASYN developing PD at a younger age and frequently suffering from an accelerated and aggressive disease course with dementia as a frequent feature^9^. These data strongly argue that ASYN plays a central role in the onset of PD and is a driver of neurodegeneration throughout the course of disease. The common clinical features and neuropathology among patients with sporadic and ASYN-driven genetic PD suggest that these types of disease converge on the ASYN aggregation pathway. Therapeutic molecules capable of preventing alpha-synuclein misfolding, without abrogating normal functions of physiologically relevant forms of ASYN would therefore be expected to provide clinical benefit for patients with both genetic and sporadic form of PD at all stages of disease. Indeed, previous studies have demonstrated the beneficial effects of treatment in vivo with interventions that directly target ASYN, including the small molecule ASYN misfolding inhibitors NPT100–18A and NPT200-11 in multiple preclinical transgenic mouse ASYN overexpression models of PD or dementia with Lewy bodies (DLB)^17–19^. Minzasolmin (UCB0599) (Fig. 1) is a single enantiomer of NPT200-11. Minzasolmin is an orally bioavailable, brain-penetrant, small molecule compound that has been evaluated in healthy participants and patients with Parkinson’s disease (ClinicalTrials.gov ID: NCT04875962^20^) and is currently in Phase 2 clinical development as a disease-modifying therapeutic for Parkinson’s disease (ClinicalTrials.gov ID: NCT04658186; EudraCT Number 2020–003265). Biophysical evaluations have revealed that UCB0599 functions at an early stage of the aggregation process by displacing membrane-bound oligomeric ASYN and returning it to a monomeric form. This in turn would prevent the formation of larger ASYN aggregates and eventually Lewy bodies^21^.

Here we describe the effects of minzasolmin on ASYN pathology and other disease-relevant endpoints in a 3-month administration study using the Line 61 transgenic mouse model of PD. This well-characterized mouse model overexpresses human wild-type ASYN and recapitulates many features of PD including peripheral and central accumulations of ASYN, evidence of neuroinflammation, dysregulated protein clearance mechanisms, and motor and non-motor dysfunction. Non-transgenic and Line 61 transgenic mice were used to evaluate the effects of minzasolmin on multiple markers of CNS pathology including ASYN burden, striatal DAT and GFAP as well as functional motor measures including grip strength and gait. Expanding on the initial efficacy studies previously conducted with NPT200-11^18^, we incorporated new analyses in the present study to investigate the relationships between reductions in neuropathology and functional outcome measures, separated and blinded analysis teams, and performed independent statistical analysis. This data package forms the basis for clinical development of the alpha-synuclein misfolding inhibitor minzasolmin as a disease-modifying therapeutic for Parkinson’s disease.

## Results

### Favorable pharmacokinetic profile of minzasolmin in C57/Bl6 mice

Minzasolmin administered intraperitoneally showed roughly linear dose-exposure proportionality at the doses tested (1 and 5 mg/kg). The compound was rapidly absorbed and the maximum plasma concentration of minzasolmin was observed at 0.25 to 0.5 h after dosing. Similarly, the highest brain levels were observed 0.5 h after dosing. Additionally, the time-brain concentration profile was parallel to the time-plasma concentration suggesting fast brain/plasma equilibrium (Supplementary Fig. 1). Brain exposure was roughly linear in the range of doses tested. The minzasolmin brain/plasma exposure (AUC) ratios for 1 and 5 mg/kg were 0.32 and 0.26, respectively, indicating that the compound crosses the blood-brain barrier. Data are presented as mean ± SEM (Table 1). Based on these findings, we elected to use both doses (1 and 5 mg/kg) in a 3-month efficacy evaluation in the Line 61 transgenic mouse model of Parkinson’s disease.

**Table 1:** Pharmacokinetic parameters of minzasolmin in C57/Bl6 mice following single dose intraperitoneal administration of 1 or 5 mg/kg.

**PLASMA**					
**Dose (mg/kg/day)**	***T***_**max**_ **(h)**	***C***_**max**_ **(ng/mL)**	**AUC**_**last**_ (h*ng/mL)	**AUC**_**inf**_ (h*ng/mL)	**Half-life (h)**
**1**	0.5	332 ± 18	307 ± 13	308	0.69
**5**	0.25	1317 ± 124	1635 ± 86	1648	0.83
**BRAIN**					
**Dose (mg/kg/day)**	***T***_**max**_ **(h)**	***C***_**max**_ **(ng/mL)**	**AUC**_**last**_ (h*ng/g)	**AUC**_**inf**_ (h*ng/g)	**Half-life (h)**
**1**	0.5	76.0 ± 4.0	93.9 ± 4.2	99.0	0.56
**5**	0.5	292 ± 30	394 ± 21	422	0.60

Data presented as the group mean ± SEM; 5 sampling timepoints × 3 mice/timepoint.

AUC_inf_ (Area under the curve from time 0 extrapolated to infinity (inf)) was used to calculate brain to plasma ratios. The density of brain homogenate was considered as 1 which is equivalent to plasma density.

### 3-month efficacy evaluation of minzasolmin in the Line 61 transgenic mouse model of Parkinson’s disease

#### Minzasolmin administration improves abnormal gait of Line 61 mice

**Fig. 2:**
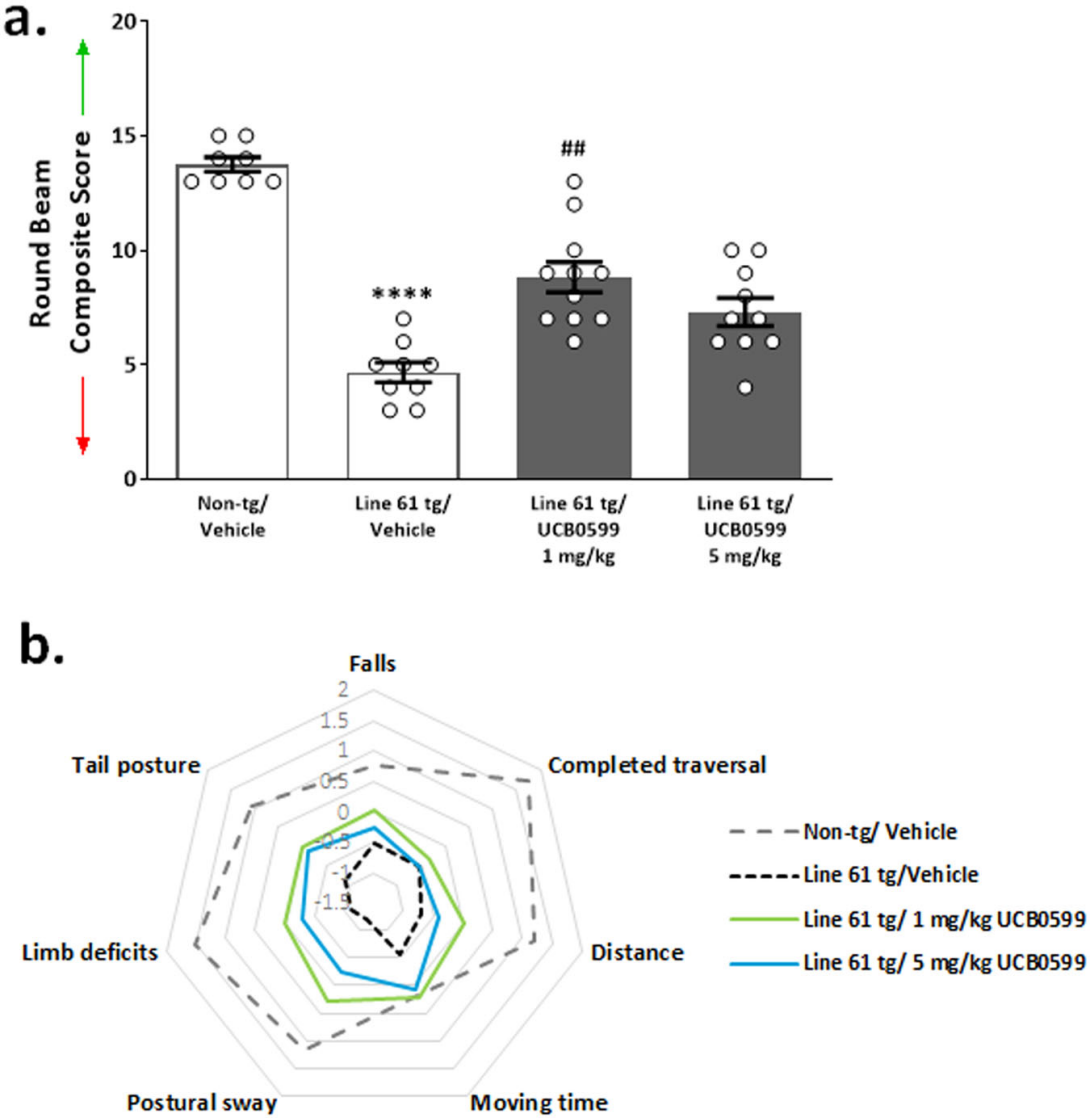
Improved gait in Line 61 transgenic mice treated with minzasolmin (UCB0599). **a** Line 61 transgenic mice had gait deficits as indicated by lower round beam performance composite scores in vehicle-treated Line 61 transgenic mice compared to non-transgenic control mice (****P < *0.0001;* N/group = 8 (Non-tg/Vehicle), 9 (Line 61 tg/Vehicle), 11 (Line 61 tg/1 mg/kg) and 10 (Line 61 tg/5 mg/kg)). Gait deficits were attenuated in 1 mg/kg minzasolmin (UCB0599)-treated Line 61 transgenic mice compared to vehicle-treated Line 61 transgenic mice with an increase in scoring (^*##*^ *P* < 0.01). Data are presented as group means ± SEM. **b** Radar plot of composite score components used to visualize the gait and balance-related deficit profile of vehicle-treated Line 61 transgenic mice compared to non-transgenic control mice that is normalized by minzasolmin treatments.

Decreased round beam composite scores were present in vehicle-treated Line 61 transgenic mice compared to non-transgenic control mice (Fig. 2a; ****P < *0*.0001; H(3) = 27.55 with a mean ranks of 34.25 for Non-tg/Vehicle, 6.611 for Line 61 tg/Vehicle, 21.77 for Line 61 tg/1 mg/kg and 16.80 for Line 61 tg/5 mg/kg), which confirms prior reporting of gait abnormalities in the Line 61 mice. Treatment with 1 mg/kg minzasolmin improved the overall scores of Line 61 transgenic mice compared to vehicle-treated Line 61 transgenic control mice (^*##*^ *P* < 0.01). Review of the scoring parameters revealed three measures with the largest apparent phenotypic differences and treatment effects including postural sway, limb deficits and tail posture (as visualized in Fig. 2b).

#### Minzasolmin administration reduces ASYN neuropathology in Line 61 mice

**Fig. 3:**
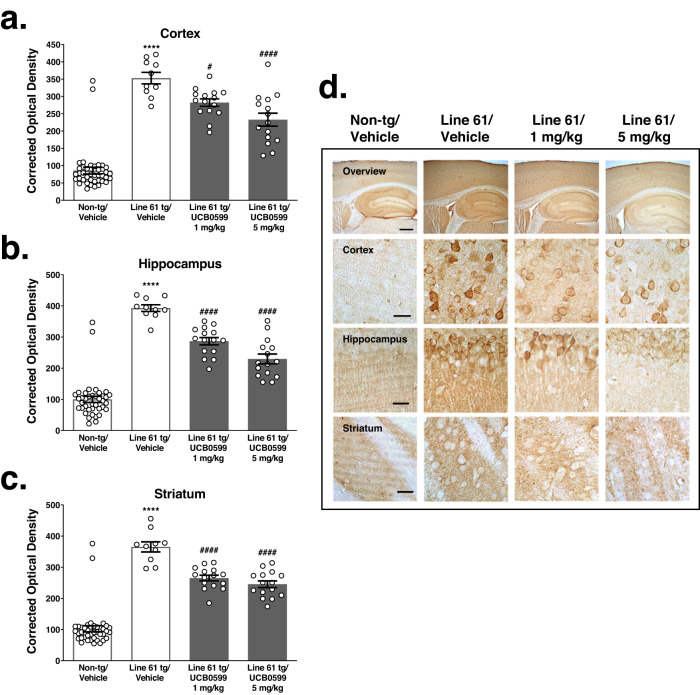
Minzasolmin (UCB0599) reduces total ASYN pathology in Line 61 transgenic mice. Statistically significant increases in total ASYN immunolabeling were observed in the (**a**) cortex, (**b**) hippocampus and (**c**) striatum of vehicle-treated Line 61 transgenic mice as compared to non-transgenic control mice (****P < 0.0001 for all regions). Compared with vehicle-treated Line 61 transgenic controls, minzasolmin (UCB0599) administration (1 and 5 mg/kg) in Line 61 transgenic mice produced statistically significant reductions in total ASYN levels in the (**a**) cortex (^*#*^ *P* < 0.05 and ^*####*^ *P* < 0.0001, respectively), (**b**) hippocampus (^*####*^ *P* < 0.0001, for both doses), and (**c**) striatum (^*####*^ *P* < 0.0001, *for both doses)*. Data are presented as group means ± SEM. Representative images in (**d**) were chosen based on the group mean for each treatment group. Scale bars represent 250 μm and 40 μm for low- and high-magnification images, respectively.

**Fig. 4:**
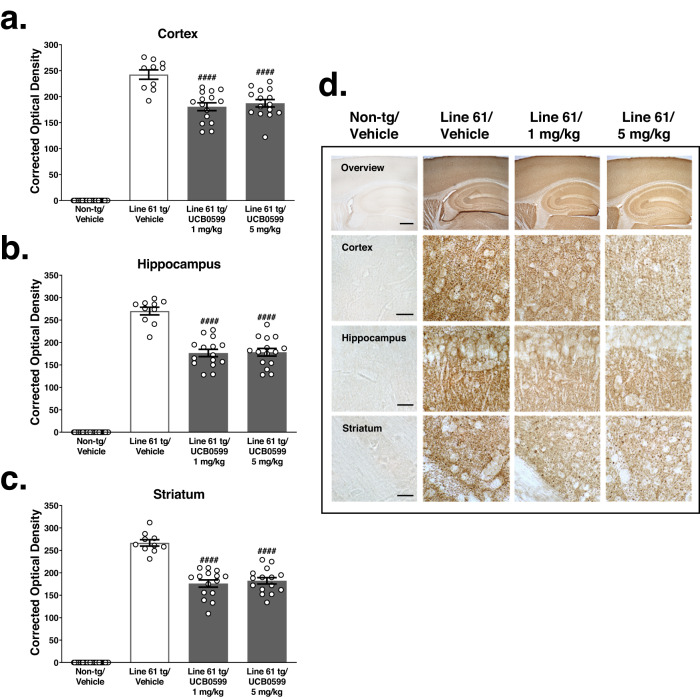
Minzasolmin (UCB0599) reduces disease-relevant proteinase K-resistant ASYN pathology in Line 61 transgenic mice. Proteinase K-resistant ASYN immunolabeling was observed in the (**a**) cortex, (**b**) hippocampus and (**c**) striatum of vehicle-treated Line 61 transgenic, but not non-transgenic control mice. Compared to vehicle-treated Line 61 transgenic controls, minzasolmin (UCB0599) administration (1 and 5 mg/kg) in Line 61 transgenic mice produced statistically significant reductions in total ASYN levels in the (**a**) cortex (**b**) hippocampus, and (**c**) striatum (^*####*^ *P* < 0.0001*, for all regions and doses)*. Data are presented as group means ± SEM. Representative images in (**d**) were chosen based on the group mean for each treatment group. Scale bars represent 250 μm and 40 μm for low- and high-magnification images, respectively.

Total and proteinase K-resistant ASYN immunolabeling were increased in the brains of Line 61 ASYN transgenic mice compared to non-transgenic vehicle-treated mice in all regions examined (****P < *0*.0001) (Figs. 3, 4). A summary of statistics for Total and proteinase K-resistant ASYN pathology are included in Supplementary Tables 1–3, respectively. There were statistically significant decreases in total ASYN immunolabeling in the cortex (Fig. 3a, ^*#*^ *P* < 0.05*)*, hippocampus (Fig. 3b, ^*####*^ *P* < 0.0001*)* and striatum (Fig. 3c, ####*P* < 0.0001) of Line 61 transgenic mice treated with 1 mg/kg minzasolmin compared to vehicle-treated Line 61 transgenic mice. Animals treated with 5 mg/kg minzasolmin also demonstrated statistically significant decreases in total ASYN immunolabeling in the cortex, hippocampus, and striatum (Fig. 3a–c, ####*P* < 0.0001*)*. Comparison of minzasolmin doses (using unpaired two tailed *t* tests) demonstrated a statistically significant effect of dose, with greater reductions in total ASYN in the cortex (*P* < 0.05*; t* = 2.277*,* df = 28) and hippocampus (*P* < 0.01*; t* = 2.898*.* df = 28) of Line 61 transgenic mice treated with 5 mg/kg minzasolmin compared to 1 mg/kg. There was no statistically significant effect of minzasolmin dose (1 vs. 5 mg/kg) on total ASYN in the striatum.

Examinations of insoluble ASYN immunolabeling in proteinase K pretreated sections revealed statistically significant decreases in proteinase K-resistant ASYN immunolabeling in the neuropil of the cortex, hippocampus, or striatum of 1 or 5 mg/kg minzasolmin-treated ASYN transgenic mice **(**Fig. 4a–c, ^*####*^ *P* < 0.0001*)* as compared to vehicle treated transgenic mice. There were no statistically significant effects of minzasolmin dose (1 vs. 5 mg/kg) on proteinase K-resistant ASYN in any examined region.

**Fig. 5:**
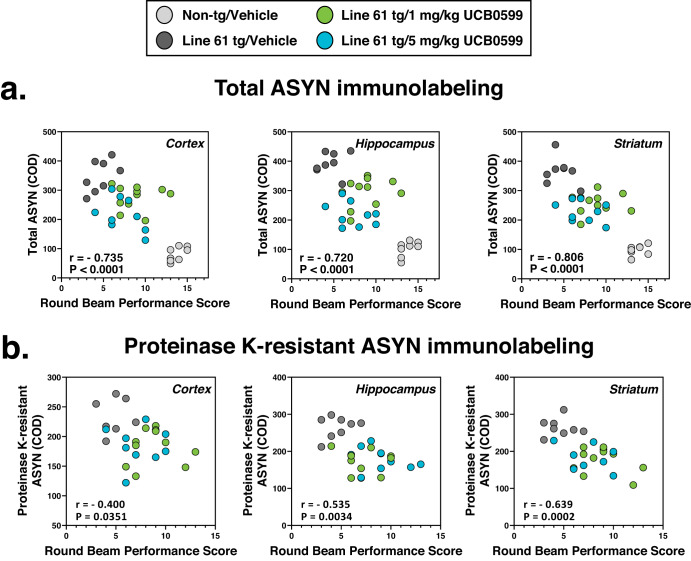
ASYN pathology is negatively correlated with round beam performance. Pearson *r* correlation analyses of (**a**) total or (**b**) Proteinase K-resistant ASYN immunolabeling versus the total round beam scoring with reveals negative correlations, demonstrating reduced round beam performance with increased ASYN burden in all regions evaluated. Accordingly, reduced ASYN pathology was associated with improved round beam performance.

Pearson’s *r* correlation coefficients were computed to assess the relationship between ASYN pathology (total or proteinase K-resistant ASYN IHC) and round beam composite score (as a measure of gait). There were negative correlations between the two variables as summarized in scatterplots presented in Fig. 5a & b, and the key statistics are presented in Table 2 demonstrating negative correlations between paired variables. There were negative correlations between total or Proteinase-K-resistant ASYN pathology burden and round beam performance scoring with increases in ASYN pathology associated with decreased round beam performance and decreased ASYN pathology associated with improved round beam performance.

**Table 2:** Summary of Pearson *r* statistics for correlation of ASYN pathology with round beam performance scoring.

ASYN measure vs. Round beam scoring	Total ASYN			Proteinase K-resistant ASYN		
Region	Cortex	Hippocampus	Striatum	Cortex	Hippocampus	Striatum
*r*	−0.735	−0.720	−0.806	−0.4	−0.535	−0.639
R squared	0.54	0.519	0.649	0.16	0.286	0.409
Number of XY Pairs	36	36	36	28	28	28
*P* Value (two-tailed)	<0.0001	<0.0001	<0.0001	0.0351	0.0034	0.0002
Confidence Interval	−0.856 to −0.535	−0.848 to −0.513	−0.897 to −0.649	−0.673 to −0.0313	−0.757 to −0.202	−0.817 to −0.350

Minzasolmin was titrated in combination with the ASYN antibody to monitor drug interference in an ASYN ELISA assay (Supplementary Figure 3). Dose-related trends in antibody binding were not observed and statistically meaningful differences between ELISA signal at different compound concentrations were not detected by ANOVA (mean ± SD, *n* = 3 per condition), demonstrating that minzasolmin does not interfere with ASYN antibody binding.

#### Minzasolmin restores dopamine transporter abundance in dorsal striatum of Line 61 transgenic mice

**Fig. 6:**
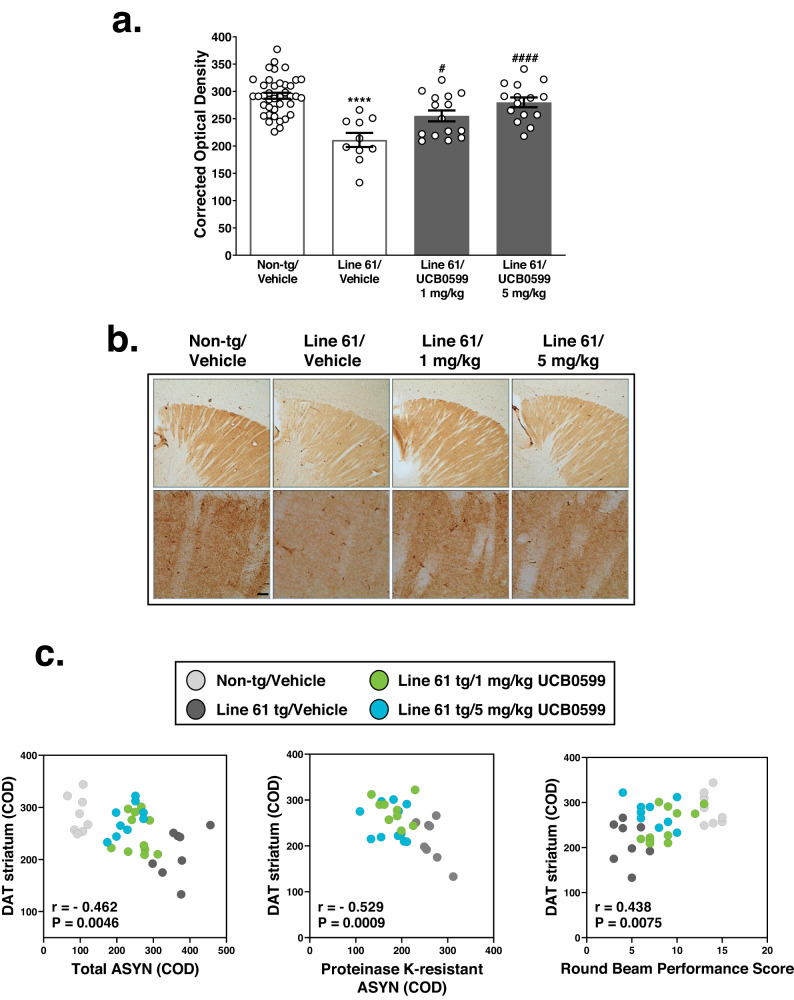
Minzasolmin (UCB0599) normalizes striatal dopamine transporter (DAT) levels in Line 61. Evaluations of dopamine transporter levels in striatum. **a** Statistically significant decreases in striatal DAT immunolabeling were observed in vehicle-treated Line 61 transgenic mice compared to non-transgenic control mice (****P < 0.0001). Compared with vehicle-treated Line 61 transgenic controls, minzasolmin (UCB0599) administration (1 and 5 mg/kg) in Line 61 transgenic mice produced statistically significant normalizations in striatal DAT levels (^*#*^ *P* < 0.05 and ^*####*^ *P* < 0.0001, respectively). Data are presented as group means ± SEM. Representative images in (**b**) were chosen based on the group mean for each treatment group. Low magnification survey images were obtained at 4x and followed by imaging at ×40 for analysis of DAT immunolabeling (scale bar = 25 mm). **c** Pearson *r* correlation analyses of DAT with (left to right panels) total or Proteinase K-resistant forms of ASYN in the striatum, or functional round beam composite scoring reveals negative and positive correlations, respectively.

A summary of statistics for the DAT immunolabeling evaluation is included in Supplementary Table 4. There were statistically significant decreases in DAT immunolabeling in the striatum of Line 61 ASYN transgenic mice compared to non-transgenic vehicle-treated control mice (****P < 0.0001) (Fig. 6a). Compared to vehicle-treated ASYN transgenic controls, 1 mg/kg and 5 mg/kg minzasolmin-treated mice demonstrated statistically significant increases in striatal DAT immunolabeling (^*#*^ *P* < 0.05 *and* ^*####*^ *P* < 0.0001). Comparison of minzasolmin dose effects demonstrated a statistical trend (*P* = 0.0754; unpaired two tailed *t* test; *t* = 1.847, df = 28) towards a greater increase in striatal DAT levels in 5 mg/kg minzasolmin -treated ASYN transgenic mice compared to 1 mg/kg.

Correlative analyses of striatal DAT levels versus ASYN pathology in the striatum or total round beam composite scoring were conducted and are summarized in Fig. 6c scatterplots. Striatal DAT levels correlated negatively with the levels in striatum of total ASYN (*r* = *−*0.462*, N* = 36*, P* = 0.00457*, CI* = *−*0.686 to −0.157*)* or proteinase K-resistant ASYN (*r* = *−*0.535*, N* = 28*, P* = 0.0033*,* CI = *−*0.757 *to −*0.203*)*, where normalized levels of DAT in the striatum were associated with lower levels of ASYN immunolabeling. Striatal DAT levels correlated positively with round beam performance score (*r* = 0.438, *N* = 36, *P* = 0.0075, CI = 0.128 to 0.670), where normalized levels of DAT in the striatum were associated with improved round beam performance scores.

#### Minzasolmin administration reduces GFAP levels, a marker of astrocyte activation, in Line 61 transgenic mice

**Fig. 7:**
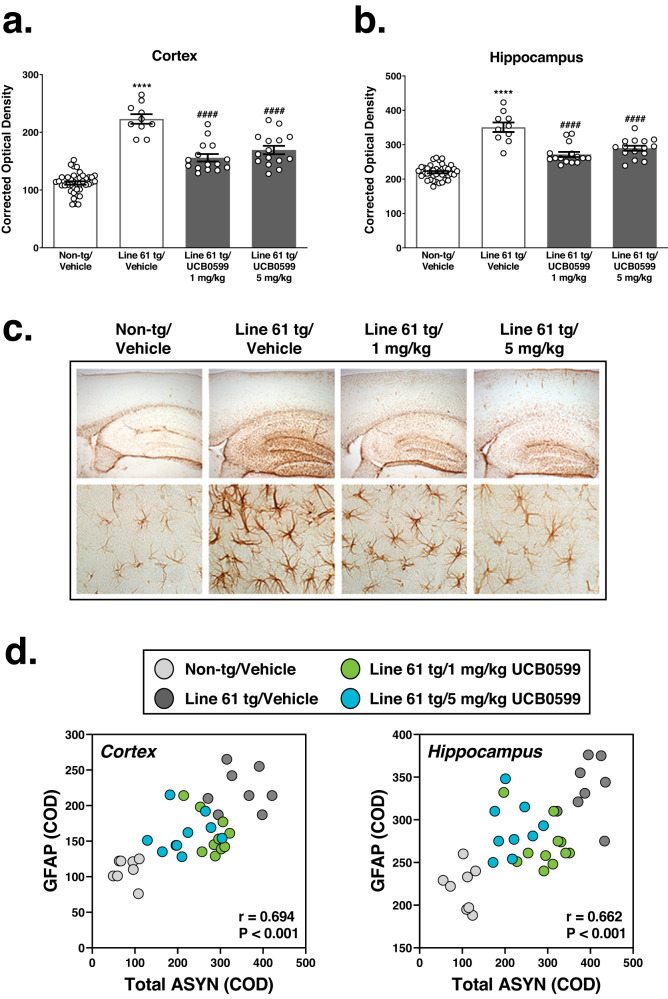
Minzasolmin (UCB0599) administration normalizes GFAP immunolabeling in Line 61 transgenic mice. GFAP immunolabeling was increased in the (**a**) neocortex and (**b**) hippocampus (CA1/2) of vehicle-treated Line 61 transgenic mice compared to non-transgenic control mice (^****^ *P* < 0.0001*, for both regions*). 1 and 5 mg/kg minzasolmin administration decreased GFAP immunolabeling in Line 61 transgenic mice compared to vehicle-treated Line 61 transgenic mice (^*####*^ *P* < 0.0001*, for both regions and doses)*. Data are presented as group means ± SEM. Representative images in (**c**) were chosen based on the group mean for each treatment group. A low-magnification overview of the hippocampus and overlying cortical regions is shown in the upper row of images. Higher-magnification inset images of GFAP immunolabeling in the hippocampus are shown in the lower row of images. **d** There were positive correlations between GFAP with total ASYN in the cortex (left panel), and the hippocampus (right panel).

A summary of statistics for the GFAP immunolabeling evaluations in cortex and hippocampus is included in Supplementary Table 5. There were statistically significant increases in GFAP immunolabeling in the cortex (Fig. 7a) and hippocampus (Fig. 7b) of Line 61 transgenic mice compared to non-transgenic vehicle-treated mice (****P < 0.0001*; both regions*). GFAP immunolabeling was decreased in the cortex (Fig. 7a, ^####^ *P* < 0.0001) and hippocampus (Fig. 7b, ^####^ *P* < 0.0001) of Line 61 transgenic mice treated with either 1 or 5 mg/kg minzasolmin compared to vehicle-treated Line 61 transgenic mice. There was no statistically significant effect of minzasolmin dose (1 vs. 5 mg/kg) on GFAP levels in either region.

Correlative analyses of GFAP levels versus ASYN pathology in cortex or hippocampus were conducted and are summarized in Fig. 7d scatterplots. GFAP levels correlated positively with the levels of total ASYN in the cortex (*r* = 0.694, *N* = 36, *P* < 0.0001, CI = 0.474 to 0.833) and hippocampus (*r* = 0.662, *N* = 36, *P* < 0.0001, CI = 0.426 to 0.814), where increased levels of GFAP were associated with increased levels of ASYN neuropathology.

#### Analysis of minzasolmin compound exposures in plasma and brain

Exposures of minzasolmin in plasma and brain were determined in plasma and brain samples obtained 1 h post final dosing of study subjects. Concentrations observed were consistent with the exposure observed in the pharmacokinetics study and confirmed brain exposure of minzasolmin. The quantified levels of minzasolmin in plasma and brain are summarized in Table 3.

**Table 3:** Analysis of levels of minzasolmin in plasma and brain samples.

Group statistics for bioanalysis of minzasolmin (group mean ± S.E.M.)			
Genotype	Treatment Group	[minzasolmin]	
		Plasma (ng/mL)	Brain (ng/g)
Non-transgenic + Line 61 transgenic	Vehicle	0 ± 0 (*N* = 51)	0 ± 0 (*N* = 52)
Line 61 transgenic mice	1 mg/kg minzasolmin	72.3 ± 7.9 (*N* = 14)	12.1 ± 1.5 (*N* = 15)
Line 61 transgenic mice	5 mg/kg minzasolmin	357.4 ± 16.8 (*N* = 14)	69.5 ± 4.7 (*N* = 15)

## Discussion

Once-daily administration of minzasolmin (UCB0599) for 3 months produced beneficial effects on disease-related ASYN neuropathology, neuroinflammation and functional motor endpoints in the Line 61 mouse model of Parkinson’s disease. These mice overexpress wild-type human ASYN and recapitulate several of the cardinal neuropathological features of PD including accumulation of aggregated ASYN, neuroinflammation, and dopamine deficits which drive motor dysfunction. We previously demonstrated lowering of total and isolated insoluble ASYN levels within 1–3 months of treatment with the racemic form of minzasolmin, NPT200-11, in Line 61 mice^18^. These prior findings were replicated and extended to minzasolmin in the current study with the key findings being that minzasolmin reduced total and insoluble (proteinase kinase-resistant) forms of ASYN in brains of Line 61 transgenic mice as assessed by immunohistochemistry. These reductions in ASYN accumulation were accompanied by reductions in markers of inflammation, neuropathology, and motor impairments. There were no apparent adverse effects of compound administration on subject health, and all treatments were well tolerated for the duration of the experiment. Analysis of compound exposures in plasma and brain from the Line 61 subject samples confirmed brain uptake of minzasolmin and were in line with the mouse pharmacokinetic evaluations conducted in wildtype C57/BL6 mice. These data informed dosing estimates for clinical studies of minzasolmin in normal healthy volunteers and patients with Parkinson’s disease^20^, in which favorable safety and pharmacokinetic profiles of minzasolmin were confirmed.

Symptomatic treatments, including pharmacological, physiotherapy and surgical approaches are available to manage the numerous motor and non-motor symptoms contributing to the decreased quality of life experienced by patients with Parkinson’s disease^22–25^. However, no disease-modifying treatments are currently available to slow, stop or reverse the progression of PD and resultant complex symptoms. Immunotherapies, antisense oligonucleotides (ASO)^26,27^ and small molecules compounds directly targeting ASYN are advancing through preclinical and clinical evaluations as putative disease-modifying therapeutics for Parkinson’s disease (*See review by* Dawson & Dawson^28^). Most current therapeutic candidates in development are monoclonal antibodies that are designed to preferentially target aggregated ASYN species hypothesized to spread from neuron to neuron thereby preventing the pathology from advancing between interconnected brain regions^29–37^. The promise of ASOs has been realized in spinal muscular atrophy^38^ and amyotrophic lateral sclerosis^39^, and much effort is being committed to exploring their use in further indications such as Multiple Systems Atrophy (NCT04165486), however, specific trials in PD have not been initiated to date. In contrast, minzasolmin is a small molecule designed to specifically inhibit the initial misfolding of membrane-bound ASYN without reducing monomeric ASYN. By intervening at the earliest stages of protein misfolding, minzasolmin prevents the feeding of misfolded ASYN into a pathological aggregation process and reduces the levels of membrane-bound oligomeric species. This would in turn also prevent later ASYN-specific pathological events including the hypothesized inter-neuronal spread of aggregated ASYN. If aggregation of ASYN is a key initiating and ongoing pathogenic event leading ultimately to progressive CNS network dysfunction with clinical motor and non-motor symptoms of Parkinson’s disease, then an intervention that unburdens the CNS from accumulating ASYN may be sufficient to slow or stop disease progression.

The heterogeneity of Parkinson’s disease patients complicates clinical trial design on many levels including patient inclusion criteria related to disease stage and choice of biomarkers to evaluate putative therapeutics. In the present study, we began treating Line 61 mice at 3 months of age, since the relevant neuropathological and motor endpoints (*i.e*., ASYN CNS pathology, reductions in striatal DAT levels, grip strength, gait dysfunction) were present or expected to emerge during the study^40^. After treating mice for 3 months with minzasolmin we observed clear reductions in ASYN pathology, which correlated with improved gait measures, restored levels of striatal DAT and reductions in GFAP (as a marker of CNS inflammation). These findings suggest several potential clinical markers that might reflect or predict therapeutic benefit of minzasolmin in patients with PD, including clinically tractable primary and exploratory endpoints (*e.g*., RT-QuiC measurements of aggregated ASYN, use of DATScan, markers of neuroinflammation in biofluids or PET imaging). Digitally-enabled evaluations of motor function are also emerging that may help differentiate patient subtypes, characterize disease progression and capture the profile of benefits exerted by disease-modifying treatments for Parkinson’s disease^41–48^.

Taken together, the current results demonstrate that treatment with the ASYN misfolding inhibitor minzasolmin reduces ASYN pathology, restores the translatable biomarker of striatal DAT, reduces neuroinflammation, and the reduced neuropathology results in normalized gait in Line 61 transgenic mice within 3 months of once-daily treatments. This comprehensive package of PD-relevant improvements supports the rationale of directly targeting misfolded ASYN with the small molecule minzasolmin as a disease-modifying strategy in Parkinson’s disease. Minzasolmin has been evaluated in Phase 1 studies assessing pharmacokinetics, brain distribution and safety and tolerability in healthy volunteers and patients with Parkinson’s disease^20^. Clinical development of minzasolmin is continuing with a Phase II interventional study in patients living with early-stage Parkinson’s disease (ClinicalTrials.gov ID: NCT04658186; EudraCT Number 2020–003265).

## Methods

### Chemicals and reagents

Minzasolmin (a single enantiomer of NPT200-11) was provided by UCB Pharma. The purity of minzasolmin was >98%. All other reagents were obtained from readily available commercial sources as listed.

### Study design

#### Mouse pharmacokinetics

Characterization of exposure profile and brain distribution of minzasolmin in male C57BL/6 mice was performed at UCB BioPharma SPRL (Braine l’Alleud, Belgium). All the procedures were approved by the UCB Ethics Committee for Animal Experimentation and conducted in compliance with the Helsinki declaration and the guidelines of the European Community Council directive 86/609/EEC. UCB BioPharma is fully accredited by the Association for Assessment and Accreditation of Laboratory Animal Care (AAALAC). Male C57BL/6 mice (8 weeks of age, 20–27 g), purchased from Charles River Laboratories (Toulouse, France), were dosed intraperitoneally with 1 or 5 mg/kg of minzasolmin (10 mL/kg of 40% w/v Captisol® in water). Three mice were used per dose and per time of sampling (0.25, 0.5, 1, 2.5, and 6 h). Mice were anaesthetized with isofluorane and blood was collected from the vena cava into K2EDTA tubes. Animals were then perfused through the left ventricle with 20 mL of heparinized NaCl 0.9% (20 UI/mL), rate flow 20 mL/min, 1 min perfusion time. At the end of the procedure mice were sacrificed by cervical dislocation. Brain samples were weighed and collected in cold glass vials and stored at −20 °C, until bioanalysis. All blood samples were thoroughly but gently mixed following collection and placed directly on ice then centrifuged at 4 °C, 3000 *g* for 15 min within 30 min after sampling. The collected plasma was stored at −20 °C, until bioanalysis. Samples were analyzed by liquid chromatography-tandem mass spectrometry (LC-MS/MS). Results of minzasolmin concentrations in plasma and brain are reported in ng/mL and ng/g respectively. Pharmacokinetic profiling was performed by non-compartmental analysis, using Phoenix WinNonlin v6.4 software (Pharsight Inc., St. Louis, MO). All computations utilized the sparse sampling method according to the experimental composite sampling regimen.

#### Animal subjects in 3-month administration study

A transgenic mouse model of Parkinson’s disease overexpressing human wild-type ASYN under the murine Thy-1 promoter on a mixed C57Bl/6 background was used in the present studies (commonly referred to Line 61)^49^. The Line 61 transgenic mouse model of Parkinson’s disease develops extensive accumulation of ASYN in areas relevant to Parkinson’s disease^49–51^, neurodegeneration (as evidenced by loss of tyrosine hydroxylase^52,53^ and dopamine transporter immunoreactivity^18^ in the striatum), inflammation^54^, and both motor and non-motor functional deficits^55^. Multiple cohorts of mice were transferred from the University of California, San Diego to a Neuropore-funded AAALAC-accredited vivarium facility per a laboratory service agreement between Neuropore Therapies, Inc. and the laboratory of Dr. Eliezer Masliah under institutional animal care and use committee (IACUC) Protocol S02221. All in vivo testing was conducted by Neuropore personnel under Explora Biosciences animal care and use protocol (ACUP) number E15-016-2. Three-month-old male transgenic and age-matched non-transgenic littermate control mice were used and housed up to 5 per cage. Animals received free access to food and water prior to the experiment. The vivarium ambient room temperature was monitored daily and kept within a range of 68–72 degrees Fahrenheit. Animals were kept on a 12:12 diurnal light cycle, with lights on at 0600.

Minzasolmin was dissolved in a vehicle solution consisting of 40% Captisol (CyDex Pharmaceuticals, Inc.; Kansas, USA) in sterile water and administered at a volume of 0.1 ml/20 g of body weight. Animals received a Monday–Friday daily intraperitoneal injection of vehicle, or 1 or 5 mg/kg minzasolmin for approximately 90 days. Animals received treatment up to and including a final injection 1 h prior to euthanasia. All study solutions were blind coded and experimenters were blinded to genotype and treatment group assignments for the duration of the study, ex vivo evaluations (i.e., neuropathology sample processing and imaging, videotape scoring) and through penultimate data analyses (i.e., data quality control with documented exclusions prior to unblinding; unblinding required for a priori pairwise comparisons).

### Statistical analyses

Statistical analyses were performed using GraphPad Prism version 9.2.0 for Windows (GraphPad Software, San Diego, California USA). Unless noted, data were analyzed by parametric one-way ANOVA with key comparisons defined a priori, to test for a phenotypic difference between vehicle-treated groups and for minzasolmin-related treatment effects in Line 61 transgenic mice. In the event of a statistically significant ANOVA (*P* < 0.05), post hoc comparisons were made using Dunnett’s multiple comparisons test with the vehicle-treated Line 61 transgenic group as the control. The criterion for statistical significance was *P* < 0.05 for all analyses. The data are presented in bar graphs as the mean ± the standard error of the mean (SEM).

### Motor behavioral assessments

Behavioral assessments were started on approximately day 70 of treatment, between the hours of 09:00 and 16:00. All behavioral assessments were conducted by Neuropore employees.

#### Grip strength

We previously reported that NPT200-11 administration did not improve progressive Line 61 grip strength deficits^18^. Nonetheless, a baseline measurement of grip strength is always included to confirm existing deficits in Line 61 transgenic mice and to inform pseudorandomized treatment group assignment (i.e., ensuring that each treatment group started with a similar distribution of grip strength performances). Mouse peak grip strength data were therefore collected at baseline (prior to starting treatments) using a grip-strength apparatus from San Diego Instruments (Poway, CA USA) as described previously^18^. Data were entered into an MS Excel spreadsheet for subsequent analysis and graphing using GraphPad Prism (GraphPad Software, Inc., La Jolla, CA USA). The dependent measure, peak strength (in grams of force), was determined per trial and as an average for each animal across trials. Data are presented (Supplementary Figure 2) across trials as group mean ± standard error of the mean (SEM). Mouse grip strength was retested following chronic treatment with minzasolmin, which confirmed previous results with NPT200-11^18^ demonstrating no statistically significant benefits of treatment on this measure (two-way ANOVA with repeated measures.

#### Round beam traversal test

Line 61 transgenic mice have robust and visually apparent deficits in gait as evaluated in the round beam traversal test by 6 months of age that are improved by treatment with small molecule compounds including ASYN misfolding inhibitors^17,18^ and the molecular tweezer compound CLR01^56^. As described previously^18^, mice were videotaped traversing a round beam apparatus after 70 days of treatment. Videotapes were then scored by a secondary evaluator using a newly developed composite rating system for specific observed features of Line 61 gait during the round beam assessment. Round beam assessments and video scoring were performed by experimenters blinded to both genotype and treatment group. A possible total composite score of 15 points included scored parameters for each subject as summarized in Table 4. A higher score indicates better performance, and a lower score indicates performance deficits. Scores were analyzed by nonparametric Kruskal–Wallis one-way ANOVA with key comparisons defined a priori, first to test for a phenotypic difference between vehicle-treated groups and then to test for minzasolmin-related treatment effects in Line 61 transgenic mice. *Post hoc* comparisons were made using Dunn’s multiple comparisons test with the vehicle-treated Line 61 transgenic group as the control.

**Table 4:** Round beam performance composite scoring parameters.

Parameter	Scoring
Falls—Did animal fall off the beam?	Yes = 0 No = 1
Did animal complete traversal?—Success criteria = 100 cm in < 60 seconds	Yes = 1 No = 0
Distance (cm)—Binned original values indexed	0–10 cm = 0 11–20 cm = 1 21–40 cm = 2 41–60 cm = 3 61–80 cm = 4 81–100 cm = 5
Moving time score—Timed amount spent moving forward, calculated as a % of total time and then indexed	0–25% = 0 26–50% = 1 51–75% = 2 76–100% = 3
Postural sway—While moving	Present and constant = 0 Present and intermittent = 1 Not present = 2
Limb deficits	Front + rear limb deficits = 0 Front OR rear limbs only = 1 No deficits = 2
Tail posture—While moving	Down/below beam = 0 Up/above beam = 1

### Neuropathological evaluations in Line 61 study samples

All subjects were euthanized within 1 h of the last treatment and brain and blood were collected. Tissue processing and imaging methods were conducted as described previously^17,18,52^ and performed under a lab service agreement between UCSD and Neuropore Therapies. All samples were blind coded to genotype and treatment group throughout immune-histological processing and imaging. The right hemibrain was post-fixed in phosphate-buffered 4% paraformaldehyde (pH 7.4) at 4 °C for 48 and then serially sectioned into 40 µM thick sagittal sections using a vibratome. Sections were free-floated and incubated overnight at 4 °C with primary antibodies. To confirm the specificity of primary antibodies, control experiments were performed in which sections were incubated overnight in the absence of primary antibody, pre-immune serum, or primary antibody pre-adsorbed for 48 h with 20-fold excess of the corresponding peptide. Tissue collection, processing, and imaging methods were conducted as described previously^49^. Briefly, the right hemibrain was post-fixed in phosphate-buffered 4% paraformaldehyde (pH 7.4) at 4 °C for 48 h, and then serially sectioned into 40 µm thick coronal sections using a vibratome. Sections were free-floated and incubated overnight at 4 °C with primary antibodies. To confirm the specificity of primary antibodies, control experiments were performed in which sections were incubated overnight in the absence of primary antibody (*data not shown*). Immunolabeled sections were imaged with a Zeiss 63× (N.A. 1.4) objective on an Axiovert 35 microscope (Zeiss, Germany). Immunolabeling was evaluated as a corrected optical density across a field of view. Summary tables of statistics for each measure are presented in Supplementary Tables 1–5.

#### Immunohistochemical evaluations of ASYN pathology

ASYN pathology in brain sections from Line 61 transgenic mice treated with minzasolmin for 3 months was evaluated via immunohistochemical evaluations of total ASYN and proteinase K-resistant ASYN. Total ASYN was immunolabeled using a mouse monoclonal anti-ASYN antibody (1:500; SYN-1, BD Biosciences, San Jose, CA). This antibody recognizes a conserved epitope in human and rodent localized to residues 91–99 of the full length ASYN which overlaps portions of the NAC and C-terminus regions of the protein and therefore immunolabels wildtype, monomeric and post translationally modified forms of ASYN such as pS129^57^. Sections were incubated overnight with the primary antibodies for evaluations of total ASYN. The next day, sections were incubated with biotinylated secondary antibodies (1:200, Vector Laboratories, Burlingame, CA USA) and visualized using an avidin-biotin (ABC) kit (Vector Laboratories) with diaminobenzidine tetrahydrochloride (DAB; Sigma-Aldrich, St. Louis, MO USA) as the chromogen. A separate set of sections were pretreated with proteinase K (#3115879001, Sigma-Aldrich) prior to a blocking step to isolate the insoluble disease-relevant forms of ASYN prior to immunolabeling as described previously^58^. Following proteinase K digestion, sections were processed as described above.

#### Assessment of drug interference by ASYN ELISA

Recombinant human ASYN was diluted in bicarbonate/carbonate coating buffer (Thermo Fisher Scientific, catalog #28382) and bound to white microtiter plates over night at 4 °C. Unbound protein was removed by repeated washing with PBS + 0.1% Tween 20. The plate was blocked for 1 h with Intercept blocking buffer (LiCor, catalog #927-70001) followed by the addition of 0.1 µg/ml SYN-1/clone 42 antibody (BD Biosciences, San Jose, CA; catalog #610786) in the presence or absence of minzasolmin. The primary antibody was incubated for 1 h at room temperature, then the plate was extensively washed with PBS + 0.1% Tween 20. The secondary anti-mouse HRP antibody (Invitrogen, A16078) was diluted 1:20,000 and allowed to incubate for 1 h at room temperature. After extensive washing, the plate was developed with SuperSignal Femto ELISA reagent (Thermo Fisher Scientific, catalog #37074) with the entire light spectrum collected on a SpectraMax Paradigm machine (Molecular Devices). Data are presented as background-subtracted percent increases over control wells lacking primary antibody (Supplementary Fig. 3).

#### Immunolabeling of striatal dopamine transporter (DAT) levels

Immunohistochemical evaluations of DAT were conducted using a rat polyclonal anti-DAT antibody (1:250; EMD Millipore, Temecula, CA, USA). Sections were incubated overnight with the primary antibody. The next day, sections were incubated with biotinylated secondary antibodies (1:100, Vector Laboratories) and visualized using an avidin-biotin (ABC) kit (Vector Laboratories) with diaminobenzidine tetrahydrochloride (DAB; Sigma-Aldrich) as the chromogen. The present study was conducted as a nested study run concurrently with shared vehicle control groups to evaluate NPT200-11 as a positive control and minzasolmin. The DAT immunolabeling results for NPT200-11 were presented in a previous publication^18^ as a standalone experiment, and the shared vehicle control group data are included in the separate analysis of the clinical stage molecule minzasolmin as shown in Fig. 6a.

#### Immunolabeling of glial fibrillary acidic protein (GFAP)

Immunohistochemical evaluations of the neuroinflammation marker GFAP in the neocortex and hippocampus (CA1/2) regions were conducted using a rabbit polyclonal anti-GFAP primary antibody (1:500, AB5804; EMD Millipore) followed by incubation with biotinylated secondary antibodies (1:100, Vector Laboratories, Burlingame) and visualized using an avidin-biotin (ABC) kit (Vector Laboratories) with diaminobenzidine tetrahydrochloride (DAB; Sigma-Aldrich) as the chromogen.

### Bioanalysis of compound levels in plasma and brain samples following long-term minzasolmin treatment

Analysis of minzasolmin by HPLC-MS/MS in plasma and brain homogenate study subject samples was conducted by PRA Health Sciences (Lenexa, KS, USA*)*. Group means were obtained using Graph Pad Prism v. 5.04 (San Diego, CA), and presented as mean concentrations of minzasolmin ± standard error of the mean (SEM) in plasma and brain homogenates.

## Supplementary information

Supplementary Information

nr-reporting-summary checklist

## Data Availability

Data from non-clinical studies is outside of UCB’s data sharing policy and is unavailable for sharing.
