# Supplementary material for: In vivo effects of the alpha-synuclein misfolding inhibitor minzasolmin supports clinical development in Parkinson’s disease
Source: NPJ Parkinsons Dis. 2023 Jul 17;9:114. doi: 10.1038/s41531-023-00552-7 (PMC10352257; doi:10.1038/s41531-023-00552-7)
Supplement: Supplementary file 1 — Supplementary Information [file 41531_2023_552_MOESM1_ESM.pdf]

SUPPLEMENTARY INFORMATION

Pharmacokinetic study of intraperitoneal administration of minzasolmin in wildtype mice

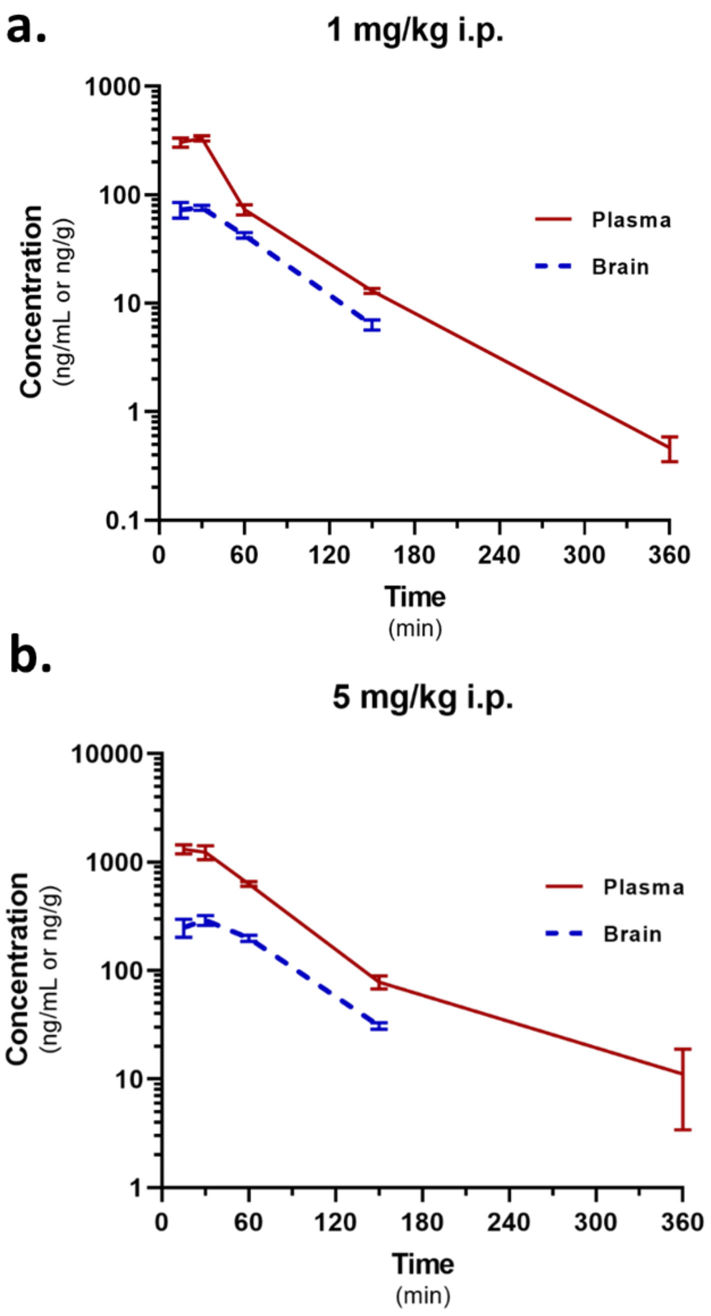

Supplementary Figure 1. Time-concentration curve profile (mean ± SEM, n=3 per time point and dose; 30 mice total) of minzasolmin observed in plasma and brain following single intraperitoneal administration of **(a)** 1 or **(b)** 5 mg/kg to male C57BL/6 mice

Evaluations of grip strength deficits of Line 61 transgenic mice

Baseline evaluations of grip strength were used to confirm a starting phenotype for Line 61 transgenic mice (*i.e.,* reduced grip strength compared to non-transgenic littermate controls) and informed pseudo randomization of subjects across treatment groups. Data are presented (**Supplementary Figure 2**) as mean ± SEM.

First, an unpaired two-tailed t-test was used to evaluate grip strength of non-transgenic and Line 61 transgenic mice prior to treatment. There was a statistically significant decrease in Line 61 transgenic grip strength compared to non-transgenic mice (**Supplementary Figure 2a**; *****P<0.0001 vs. non-transgenic mice; t=11.29, df=84*), confirming that the Line 61 transgenic mice had existing grip strength deficits prior to study treatments.

To ensure appropriate distribution of subjects across treatment groups, evaluation of baseline grip strength was re-analyzed following treatment group assignment using a one-way ANOVA. Specific exclusions related to post-study genotype confirmation were documented and the group numbers for all baseline grip strength analyses were: Non-tg/Vehicle (39), Line 61 tg/Vehicle (14), Line 61 tg/1 mg/kg minzasolmin (16) and Line 61 tg/5 mg/kg minzasolmin (17). There was a statistically significant difference in grip strength of vehicle-treated Line 61 transgenic compared to non-transgenic mice (**Supplementary Figure 2b**; ****P<0.0001 vs. non-transgenic mice*). There were no statistically significant differences between the vehicle and minzasolmin - treated Line 61 transgenic groups, indicating an appropriate treatment group distribution of transgenic subjects.

Grip strength was reassessed following treatments with vehicle or minzasolmin (**Supplementary Figure 2c**) using a two-way repeated measures ANOVA. Specific exclusions related to genotype confirmation, health and test issues, and study deaths prior to re-test were documented. The group numbers for this analysis were: Non-tg/Vehicle (39), Line 61 tg/Vehicle (11), Line 61 tg/1 mg/kg minzasolmin (14) and Line 61 tg/5 mg/kg minzasolmin (13). There was a statistically significant interaction of treatment group x time (P=0.0149; F (7,73) = 3.727), and *post hoc* comparisons via Dunnett's multiple comparisons test revealed statistically significant differences in grip strength between vehicle-treated Line 61 transgenic compared to non-transgenic mice at baseline and re-test (***P<0.01 vs. non-transgenic mice*). There were no statistically significant differences between the vehicle-treated Line 61 transgenic group and any other transgenic treatment group.

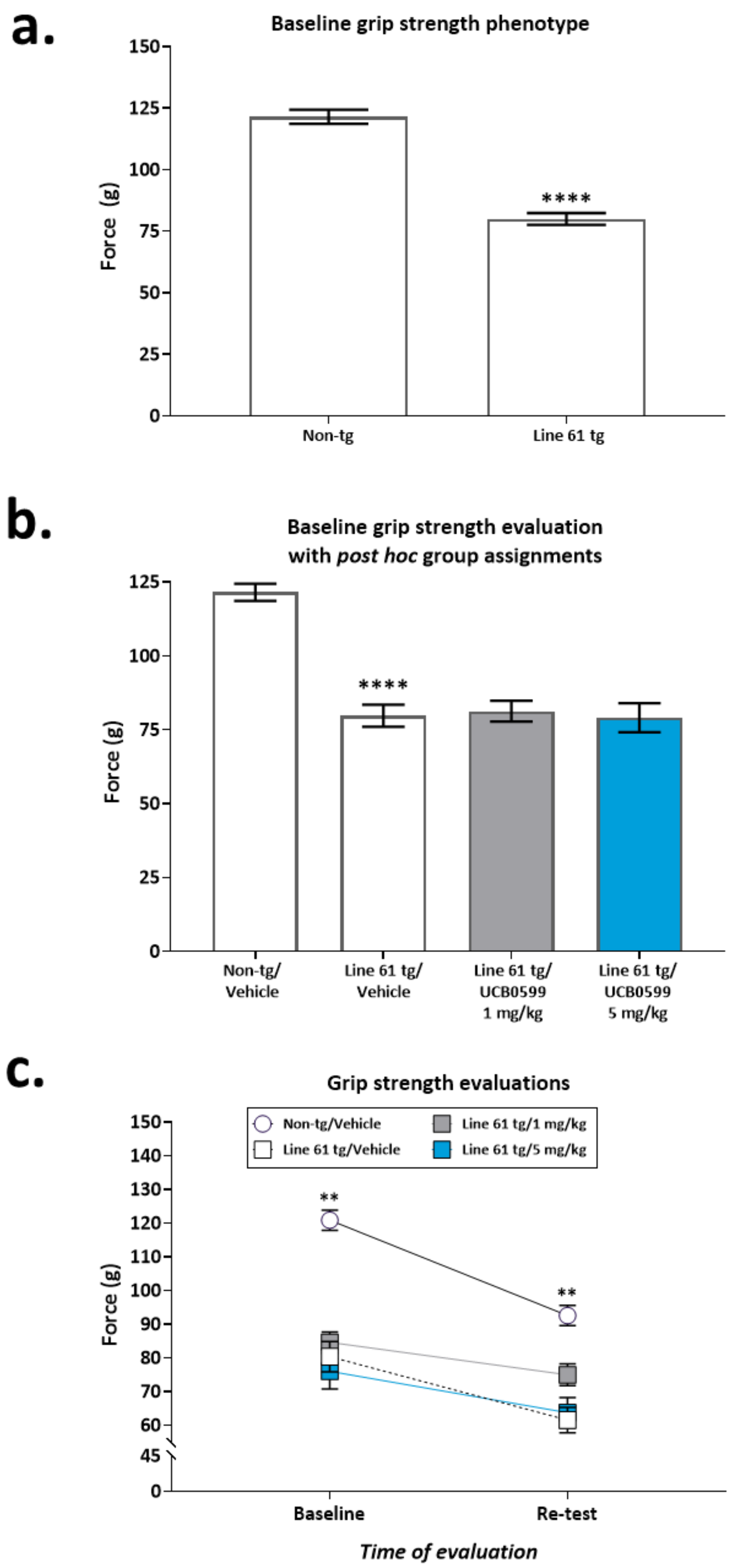
a.
Baseline grip strength phenotype
Force (g)
150
125
100
75
50
25
0
****
Non-tg
Line 61 tg
b.
Baseline grip strength evaluation
with *post hoc* group assignments
Force (g)
125
100
75
50
25
0
****
Non-tg/
Vehicle
Line 61 tg/
Vehicle
Line 61 tg/
UCB0599
1 mg/kg
Line 61 tg/
UCB0599
5 mg/kg
c.
Grip strength evaluations
Non-tg/Vehicle
Line 61 tg/1 mg/kg
Line 61 tg/Vehicle
Line 61 tg/5 mg/kg
Force (g)
150
140
130
120
110
100
90
80
70
60
45
0
**
**
Baseline
Re-test
Time of evaluation

Supplementary Figure 2. Baseline and post-treatment evaluations of Line 61 grip strength deficits

Baseline evaluations of grip strength prior to starting treatments were used to **(a)** confirm grip strength deficits in Line 61 transgenic mice compared to non-transgenic mice (*****P<0.0001 vs. non-transgenic mice)* and **(b)** ensure the appropriate distribution of subjects across assigned treatment groups (****P<0.0001 Line 61 tg vs. non-transgenic vehicle-control mice*); there were no statistically significant differences between the vehicle and minzasolmin-treated Line 61 transgenic groups). Post-treatment reassessment of grip strength **(c)** revealed statistically significant differences in grip strength between vehicle-treated Line 61 transgenic compared to non-transgenic mice at baseline and re-test (***P<0.01 vs. non-transgenic mice*). There were no statistically significant differences between the vehicle-treated Line 61 transgenic group and any other transgenic treatment group. Data are presented as mean ± SEM.

Assessment of drug interference by ASYN ELISA

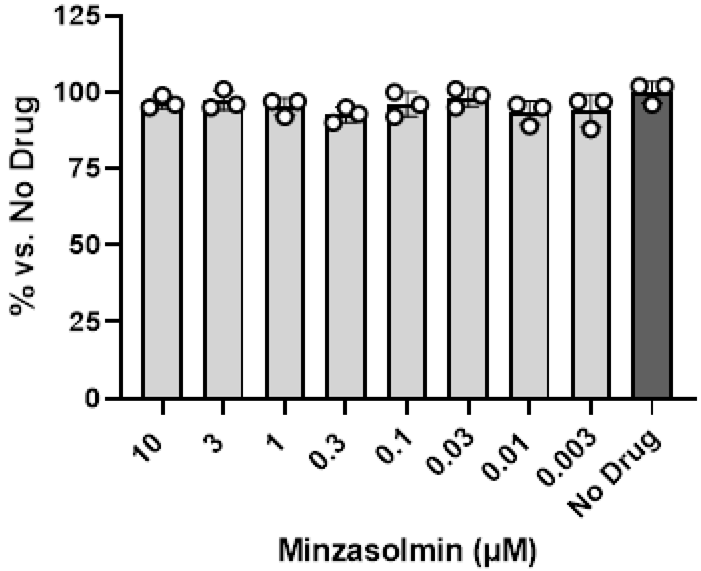

Supplementary Figure 3. Minzasolmin does not interfere with SYN-1 antibody binding
Minzasolmin was titrated in combination with the SYN-1 antibody to monitor drug interference in an ELISA assay. Dose-related trends in antibody binding were not observed and statistically meaningful differences between ELISA signal at different compound concentrations were not detected by ANOVA (mean ± SD, n=3 per condition).

SUPPLEMENTARY TABLES

Supplementary Table 1. Summary of statistics for neuropathological endpoints

Pathology Endpoint	Region	Main ANOVA P Value	F	Degrees of Freedom (DFn, DFd)	R^2
**Total ASYN**	**Cortex**	<0.0001	78.51	3,75	0.7585
	**Hippocampus**	<0.0001	93.51	3,75	0.7891
	**Striatum**	<0.0001	87.32	3,75	0.7774
**Proteinase K+ ASYN**	**Cortex**	<0.0001	15.96	2, 37	0.4631
	**Hippocampus**	<0.0001	33.71	2, 37	0.6457
	**Striatum**	<0.0001	37.56	2, 37	0.67
**DAT**	**Striatum**	<0.0001	14.68	3, 75	0.3699
**GFAP**	**Cortex**	<0.0001	74.23	3, 75	0.7481
	**Hippocampus**	<0.0001	70.43	3, 75	0.738

Supplementary Table 2. Summary of statistics for Total ASYN pathology

	Region	Mean	SEM	N	Adjusted P Value (vs. control group)	Confidence interval of mean
**Non-tg/ Vehicle**	**Cortex**	85.64	9.887	39		65.63 to 105.7
	**Hippocampus**	100.0	9.908	39		79.97 to 120.1
	**Striatum**	102.4	9.927	39		82.31 to 122.5
**Line 61 tg/ Vehicle**	**Cortex**	352.8	16.60	10	<0.0001	315.3 to 390.3
	**Hippocampus**	392.9	10.70	10	<0.0001	368.7 to 417.1
	**Striatum**	365.3	16.22	10	<0.0001	328.6 to 402.0
**Line 61 tg/ 1 mg/kg minzasolmin**	**Cortex**	282.1	10.75	15	0.0148	259.1 to 305.2
	**Hippocampus**	286.7	11.79	15	<0.0001	261.5 to 312.0
	**Striatum**	265.4	9.001	15	<0.0001	246.1 to 284.7
**Line 61 tg/ 5 mg/kg minzasolmin**	**Cortex**	232.8	18.81	15	<0.0001	192.5 to 273.1
	**Hippocampus**	229.7	15.78	15	<0.0001	195.8 to 263.5
	**Striatum**	245.5	10.74	15	<0.0001	222.4 to 268.5

Supplementary Table 3. Summary of statistics for Proteinase K-resistant ASYN pathology

Group	Region	Mean	SEM	N	Adjusted P Value (vs. control group)	Confidence interval of mean
**Line 61 tg/ Vehicle**	**Cortex**	242.5	9.062	10		222.0 to 263.0
	**Hippocampus**	270.1	8.574	10		250.7 to 289.5
	**Striatum**	266.7	7.079	10		250.7 to 282.7
**Line 61 tg/ 1 mg/kg minzasolmin**	**Cortex**	180.6	7.575	15	<0.0001	164.4 to 196.8
	**Hippocampus**	176.6	8.087	15	<0.0001	159.3 to 193.9
	**Striatum**	176.1	7.989	15	<0.0001	159.0 to 193.3
**Line 61 tg/ 5 mg/kg minzasolmin**	**Cortex**	187.3	7.153	15	<0.0001	172.0 to 202.7
	**Hippocampus**	178.3	8.452	15	<0.0001	160.1 to 196.4
	**Striatum**	182.2	7.081	15	<0.0001	167.0 to 197.4

Supplementary Table 4. Summary of statistics for DAT immunolabeling evaluation in dorsal striatum

Group	Mean	SEM	N	Adjusted P Value (vs. control group)	Confidence interval of mean
**Non-tg/ Vehicle**	292.1	5.555	39		280.9 to 303.3
**Line 61 tg/ Vehicle**	211.2	12.90	10	<0.0001	182.0 to 240.4
**Line 61 tg/ 1 mg/kg minzasolmin**	255.3	10.03	15	0.0117	233.8 to 276.8
**Line 61 tg/ 1 mg/kg minzasolmin**	280.1	8.932	15	<0.0001	260.9 to 299.2

Supplementary Table 5. Summary of statistics for GFAP immunolabeling evaluations

Group	Region	Mean	SEM	N	Adjusted P Value (vs. control group)	Confidence interval of mean
**Non-tg/ Vehicle**	**Cortex**	112.2	2.882	39		106.3 to 118.0
	**Hippocampus**	220.1	3.319	39		213.4 to 226.8
**Line 61 tg/ Vehicle**	**Cortex**	223.1	8.295	10	<0.0001	204.3 to 241.9
	**Hippocampus**	350.8	13.91	10	<0.0001	213.4 to 226.8
**Line 61 tg/ 1 mg/kg minzasolmin**	**Cortex**	155.9	6.469	15	<0.0001	142.0 to 169.7
	**Hippocampus**	271.6	7.448	15	<0.0001	255.6 to 287.6
**Line 61 tg/ 1 mg/kg minzasolmin**	**Cortex**	169.3	7.241	15	<0.0001	153.8 to 184.9
	**Hippocampus**	289.7	7.337	15	<0.0001	273.9 to 305.4
