# Supplementary material for: In vivo effects of the alpha-synuclein misfolding inhibitor minzasolmin supports clinical development in Parkinson’s disease
Source: NPJ Parkinsons Dis. 2023 Jul 17;9:114. doi: 10.1038/s41531-023-00552-7 (PMC10352257; doi:10.1038/s41531-023-00552-7)
Supplement: Supplementary file 2 — nr-reporting-summary checklist [file 41531_2023_552_MOESM2_ESM.pdf]

nature portfolio

Corresponding author(s): Diana Price (on behalf of Martin Citron)

Last updated by author(s): Jun 14, 2023

Reporting Summary

Nature Portfolio wishes to improve the reproducibility of the work that we publish. This form provides structure for consistency and transparency in reporting. For further information on Nature Portfolio policies, see our Editorial Policies and the Editorial Policy Checklist.

Statistics

For all statistical analyses, confirm that the following items are present in the figure legend, table legend, main text, or Methods section.

n/a	Confirmed	
☐	☒	The exact sample size (*n*) for each experimental group/condition, given as a discrete number and unit of measurement
☒	☐	A statement on whether measurements were taken from distinct samples or whether the same sample was measured repeatedly
☐	☒	The statistical test(s) used AND whether they are one- or two-sided<br>*Only common tests should be described solely by name; describe more complex techniques in the Methods section.*
☐	☒	A description of all covariates tested
☐	☒	A description of any assumptions or corrections, such as tests of normality and adjustment for multiple comparisons
☐	☒	A full description of the statistical parameters including central tendency (e.g. means) or other basic estimates (e.g. regression coefficient) AND variation (e.g. standard deviation) or associated estimates of uncertainty (e.g. confidence intervals)
☐	☒	For null hypothesis testing, the test statistic (e.g. *F*, *t*, *r*) with confidence intervals, effect sizes, degrees of freedom and *P* value noted<br>*Give P values as exact values whenever suitable.*
☒	☐	For Bayesian analysis, information on the choice of priors and Markov chain Monte Carlo settings
☒	☐	For hierarchical and complex designs, identification of the appropriate level for tests and full reporting of outcomes
☐	☒	Estimates of effect sizes (e.g. Cohen's *d*, Pearson's *r*), indicating how they were calculated

Our web collection on statistics for biologists contains articles on many of the points above.

Software and code

Policy information about availability of computer code

Data collection	No software or code was used to collect data in this study.
Data analysis	GraphPad Prism version 9.2.0 for Windows, (GraphPad Software, San Diego, California USA) was used for statistical analysis and graphing.

For manuscripts utilizing custom algorithms or software that are central to the research but not yet described in published literature, software must be made available to editors and reviewers. We strongly encourage code deposition in a community repository (e.g. GitHub). See the Nature Portfolio guidelines for submitting code & software for further information.

Data

Policy information about availability of data

All manuscripts must include a data availability statement. This statement should provide the following information, where applicable:

- Accession codes, unique identifiers, or web links for publicly available datasets
- A description of any restrictions on data availability
- For clinical datasets or third party data, please ensure that the statement adheres to our policy

Data and materials availability: Reasonable requests for access to data generated and analyzed in these studies will be considered by the corresponding (M.C.) and/or senior author (A.L.B.).

Human research participants

Policy information about studies involving human research participants and Sex and Gender in Research.

Reporting on sex and gender	This study did not use human research participants.
Population characteristics	*Describe the covariate-relevant population characteristics of the human research participants (e.g. age, genotypic information, past and current diagnosis and treatment categories). If you filled out the behavioural & social sciences study design questions and have nothing to add here, write "See above."*
Recruitment	*Describe how participants were recruited. Outline any potential self-selection bias or other biases that may be present and how these are likely to impact results.*
Ethics oversight	*Identify the organization(s) that approved the study protocol.*

Note that full information on the approval of the study protocol must also be provided in the manuscript.

Field-specific reporting

Please select the one below that is the best fit for your research. If you are not sure, read the appropriate sections before making your selection.

☒ Life sciences ☐ Behavioural & social sciences ☐ Ecological, evolutionary & environmental sciences

For a reference copy of the document with all sections, see nature.com/documents/nr-reporting-summary-flat.pdf

Life sciences study design

All studies must disclose on these points even when the disclosure is negative.

Sample size	Neuropore has established and maintains a colony of Line 61 transgenic mice, and we have carefully characterized the mice in our colony (confirming and extending multiple aspects of the phenotype of the line). Our initial use of the model in pilot evaluations was guided by existing reports where power analysis of multimodal endpoints (Chesselet et al., 2012 - DOI: 10.1007/s13311-012-0104-2) determined that small group sizes (2-20 animals/group) were sufficient for characterizing the phenotype and detecting treatment effects. Based on our experience with the model, 8-15 mice per group has proven sufficient.
Data exclusions	All datasets are QC'd prior to unblinding with regard to any health -related issues, sample issues (damaged, ID, missing). There were no data exclusions for the neuropathology endpoints.
Replication	The present study with minzasolmin (UCB0599) was the penultimate evaluation for the alpha-synuclein misfolding inhibitor discovery program to inform clinical candidate selection and dose determinations. The results confirmed previous findings with the racemate (NPT200-11) from multiple 1 and 3 month efficacy evaluations. No further in vivo evaluations (in the rodent model) were conducted once UCB0599 was in clinical development.
Randomization	For our studies with the Line 61, a baseline measurement of grip strength is always included to confirm existing deficits in Line 61 transgenic mice and to inform pseudorandomized treatment group assignment (i.e., ensuring that each treatment group started with a similar distribution of grip strength performances).
Blinding	All study solutions were blind coded and experimenters were blinded to genotype and treatment group assignments for the duration of the study, ex vivo evaluations (i.e., neuropathology sample processing and imaging, videotape scoring) and through penultimate data analyses (i.e., data quality control with documented exclusions prior to unblinding; unblinding required for apriori pairwise comparisons).

Reporting for specific materials, systems and methods

We require information from authors about some types of materials, experimental systems and methods used in many studies. Here, indicate whether each material, system or method listed is relevant to your study. If you are not sure if a list item applies to your research, read the appropriate section before selecting a response.

Materials & experimental systems

n/a	Involved in the study
☐	☒ Antibodies
☒	☐ Eukaryotic cell lines
☒	☐ Palaeontology and archaeology
☐	☒ Animals and other organisms
☒	☐ Clinical data
☒	☐ Dual use research of concern

Methods

n/a	Involved in the study
☒	☐ ChIP-seq
☒	☐ Flow cytometry
☒	☐ MRI-based neuroimaging

Antibodies

Antibodies used

Used in evaluations of samples from 3 month efficacy study (outsourced to provider; lot numbers not available)
Total ASYN pathology: mouse monoclonal anti-ASYN antibody (1:500; SYN-1/Clone 42,catalog # 610786 -BD Biosciences, San Jose, CA).
Dopamine transporter: rat polyclonal anti-DAT antibody (1:250; MAB369 EMD Millipore, Temecula, CA, USA).
GFAP: rabbit polyclonal anti-GFAP primary antibody (1:500, AB5804; EMD Millipore)

Antibody used in antibody-compound interference assessment (Supplemental figure 3):
SYN-1/Clone 42,catalog # 610786 -BD Biosciences, San Jose, CA.

Validation

Neuropathological evaluations in blindcoded Line 61 study samples from the 3 month administration study were outsourced to a provider who provided a general statement, "To confirm the specificity of primary antibodies, control experiments were performed in which sections were incubated overnight in the absence of primary antibody."

Neuropore no longer outsources IHC/IF evaluations and continues to successfully use these antibodies for evaluations in the Line 61. Information from antibody-specific product pages and/or literature regarding validation/characterization (note: all antibodies have a "research use only" regulatory designation):

1. mouse monoclonal anti-ASYN antibody (SYN-1/Clone 42; # 610786)
   a. Reported mapping & specificity in mouse brain and cell lines (Perrin et al., 2003 - Neurosci. Letters)
   b. Mapped to ASYN structure (Schmid et al., 2013 - Molecular & Cellular Proteomics)

2. rat polyclonal anti-DAT antibody (MAB369)
   a. Validated for use in IC, IHC & WB use (per product page at EMD Millipore) in human and mouse tissues
   b. appropriate pattern of staining in basal ganglia shown in multiple references (listing on product page at EMD Millipore).

3. rabbit polyclonal anti-GFAP primary antibody (AB5804)
   a. Extensive listing of product citations on EMD Millipore product page

Animals and other research organisms

Policy information about studies involving animals; ARRIVE guidelines recommended for reporting animal research, and Sex and Gender in Research

Laboratory animals

Mouse Pharmacokinetic studies: Male C57BL/6 mice (8 weeks of age, 20-27g), purchased from Charles River Laboratories
3 Month Efficacy Evaluation: A transgenic mouse model of Parkinson's disease overexpressing human wild-type ASYN under the murine Thy-1 promoter on a mixed C57Bl/6 background was used in the present studies. 3 month old male transgenic and age-matched littermate non-transgenic littermate control mice were used.

Wild animals

This study did not involve wild animals.

Reporting on sex

Male mice were used in all studies. We used male Line 61 transgenic mice for the 3 month efficacy study because:
(1) male Line 61 tg mice have been extensively characterized (previous reports in literature with independent in-house characterizations for confirmation and elaboration).
(2) male Line 61 tg mice have a more robust phenotype (neuropathological features AND motor impairment).
(3) female Line 61 mice express lower levels of the human α-synuclein transgene because it is inserted in the X chromosome, resulting in random chromosomal inactivation (Chesselet et al., 2012) and high levels of variability.

Field-collected samples

The study did not involve studies with field-collected samples.

Ethics oversight

Mouse Pharmacokinetic studies conducted at UCB Pharma: All the procedures were conducted in compliance with the Helsinki declaration and the guidelines of the European Community Council directive 86/609/EEC. UCB BioPharma is fully accredited by the Association for Assessment and Accreditation of Laboratory Animal Care (AAALAC).
3 month mouse efficacy evaluation: All in vivo testing was conducted by Neuropore personnel at an AAALAC-accredited vivarium facility (Explora Biosciences) under Explora Biosciences animal care and use protocol (ACUP) number E15-016-2 .

Note that full information on the approval of the study protocol must also be provided in the manuscript.
